# Current evidence shows no influence of women's menstrual cycle phase on acute strength performance or adaptations to resistance exercise training

**DOI:** 10.3389/fspor.2023.1054542

**Published:** 2023-03-23

**Authors:** Lauren M. Colenso-Semple, Alysha C. D'Souza, Kirsty J. Elliott-Sale, Stuart M. Phillips

**Affiliations:** ^1^ Department of Kinesiology, McMaster University, Hamilton, ON, Canada; ^2^ Institute of Sport, Manchester Metropolitan University, Manchester, United Kingdom

**Keywords:** menstrual cycle, resistance training, hypertrophy, strength, exercise performance

## Abstract

**Introduction:**

The bias towards excluding women from exercise science research is often due to the assumption that cyclical fluctuations in reproductive hormones influence resistance exercise performance and exercise-induced adaptations.

**Methods:**

Hence, the purpose of this umbrella review was to examine and critically evaluate the evidence from meta-analyses and systematic reviews on the influence of menstrual cycle phase on acute performance and chronic adaptations to resistance exercise training (RET).

**Results:**

We observed highly variable findings among the published reviews on the ostensible effects of female sex hormones on relevant RET-induced outcomes, including strength, exercise performance, and hypertrophy.

**Discussion:**

We highlight the importance of comprehensive menstrual cycle verification methods, as we noted a pattern of poor and inconsistent methodological practices in the literature. In our opinion, it is premature to conclude that short-term fluctuations in reproductive hormones appreciably influence acute exercise performance or longer-term strength or hypertrophic adaptations to RET.

## 1. Introduction

Muscular adaptations to exercise involve numerous interrelated cellular and physiological mechanisms (1), some of which are influenced by biological sex. While both men and women gain muscle size and strength in response to resistance exercise training (RET), it has been hypothesized that sex may confer divergent hypertrophic potential due to hormonal differences (2). While sex hormone levels are relatively stable in men from day to day, they vary throughout the menstrual cycle in naturally cycling women (3). Given the potential complexity associated with accounting for hormonal fluctuations in women, there is a bias towards the inclusion of men and the exclusion of female participants in exercise science research (4, 5). Thus, although the extent to which sex-specific factors influence RET-induced muscular adaptations is still unclear, researchers and practitioners often assume that the results of interventions in men are equally applicable to women (6).

While the anabolic effects of androgenic hormones (primarily testosterone) are well-documented (7), the influence of ovarian hormones (primarily estradiol and progesterone) on muscle mass regulation is less clear (8, 9). Although further research is required to elucidate the underlying mechanisms, estrogen signaling may influence some pathways and processes that influence RET muscular adaptations, including protein turnover, myosin function, and satellite cell activity (8, 10, 11). The effects of estrogen deficiency have been studied primarily in ovariectomized rodent models (11), and the mechanisms by which progesterone could regulate muscle mass are largely unknown (12).

Women who are pre- and post-menopausal are also thought to be a model of the influence of estrogen on muscle adaptations; however, the menopausal transition in women is not abrupt (as in ovariectomized rodents) and shows large interpersonal variability (13). Estradiol and progesterone levels decline during menopause, and post-menopausal women are particularly susceptible to losing skeletal muscle mass and strength (14). Estrogen deficiency and the downregulation of estrogen receptor signaling appear to reduce the number of satellite cells in skeletal muscle (9, 11), potentially accelerating the loss of muscle mass and strength in post-menopausal women. Ovariectomized rodent models support this evidence, but these models do not reflect the gradual and non-linear hormonal decline that occurs with menopause (15). Thus, rodent data need to be interpreted cautiously since the extent that these data translate to human skeletal muscle is unknown. Further, given the interaction between the ovarian hormones, it is unclear how the presence of progesterone contributes to or attenuates the age-related loss of skeletal muscle (14).

**Figure 1:**
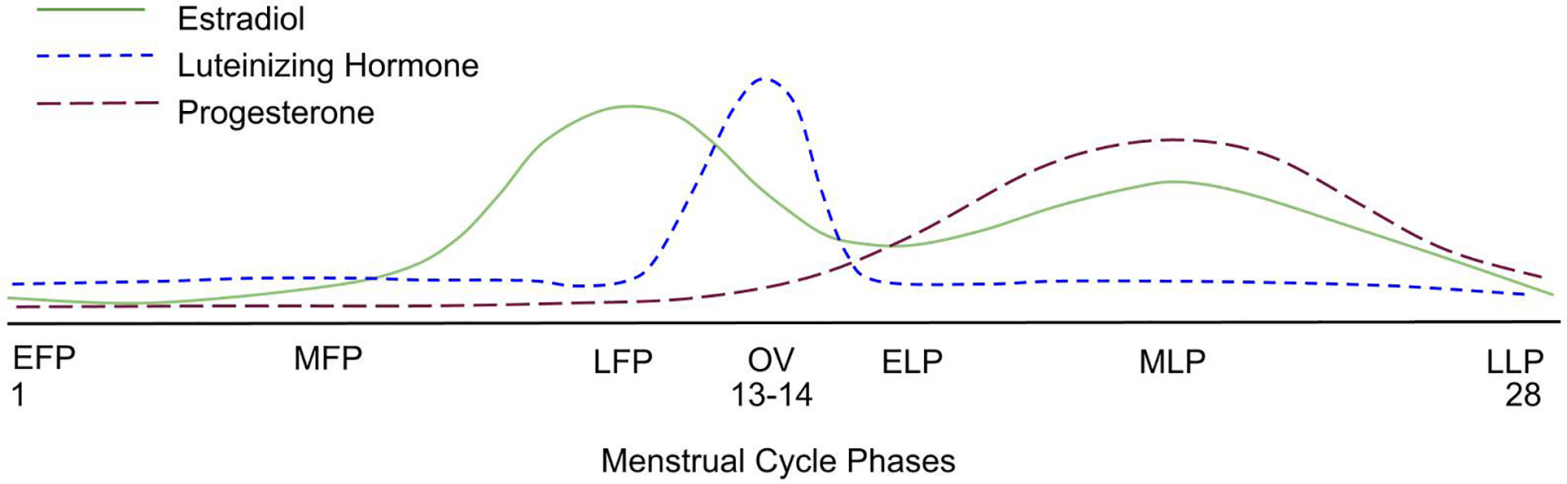
A graphical representation of the average hormonal changes that occur during a menstrual cycle, showcasing the expected rise and fall of key hormones. EFP, early follicular phase; MFP, mid-follicular phase; LFP, late follicular phase; OV, ovulation; ELP, early luteal phase; MLP, mid-luteal phase; LLP, late luteal phase.

While hormonal levels decline steadily post-menopause over many months-years, premenopausal women's physiology is unique in that hormone levels fluctuate throughout each menstrual cycle. Estrogen levels peak during the late follicular phase before ovulation, while progesterone levels are highest post-ovulation during the mid-luteal phase (Figure 1). While we acknowledge that a long-term decline in hormonal levels is likely detrimental, hormonal fluctuations throughout the menstrual cycle occur over several days. Is the short-term influence of these hormones so profound that fluctuations influence acute performance and long-term muscular adaptations to RET? Several meta-analyses (16–18) and systematic reviews (19, 20) have evaluated the influence of menstrual cycle phase on acute exercise performance and chronic adaptations to RET. While some authors report that resistance exercise performance is enhanced when estrogen is higher (i.e., late follicular phase), others conclude that there is no difference between the various menstrual cycle phases. Variable findings among the published studies are likely a result of poor and inconsistent methodological practices. Furthermore, few papers seem to be driving the opinion that follicular phase-based training (training with a higher frequency and greater volume during the follicular phase) may be superior to luteal-based and/or even more conventional (i.e., training in both phases of the menstrual cycle) forms of training (21, 22).

In this review, we aimed to fill some knowledge gaps related to how menstrual cycle phase might affect exercise performance and gains in muscle size and strength. We conducted an umbrella review of pertinent meta-analyses and systematic reviews to critically evaluate and summarize the current state of knowledge on the impact of menstrual cycle phase on resistance exercise performance and RET-induced anabolic adaptations. We included a comprehensive methodological assessment of often cited papers supporting the concept that menstrual cycle phase-based training is an effective practice.

## 2. Methods

### 2.1. Search strategy

A systematic electronic literature search was conducted in Ovid Medline, Embase, PubMed, and SportDiscus in January 2022. The search was restricted to English-language systematic reviews and meta-analyses of menstrual cycle phase-based resistance exercise training outcomes in young, healthy eumenorrheic women with no known menstrual cycle dysfunctions who were not using hormonal contraceptives. The following combination of search terms was used: (menstrual cycle OR menstrual phase OR follicular phase OR luteal phase OR estrogen OR estradiol OR hormones) AND (resistance training OR weight training OR strength training OR resistive exercise OR concentric exercise OR eccentric exercise OR force OR torque OR power OR muscular performance OR athletic performance OR sports performance). The quality of each systematic review was scored according to the 11-item A Measurement Tool to Assess Systematic Reviews (AMSTAR) tool (23). Standardized effectiveness statements (SES; i.e., sufficient evidence, some evidence, insufficient evidence, insufficient evidence to determine) were generated, and the quality of evidence (QoE) from each review was rated based on the Grading of Recommendations Assessment, Development and Evaluation (GRADE) approach (24), which considers design and AMSTAR score.

### 2.2. Results

The original search was performed in January 2022 and yielded 12 articles. Of these, 7 were excluded upon title abstract screening, yielding 5 systematic reviews and meta-analyses relevant to our research question (16–20). Two authors independently screened all reviews (ACD and LCS), and a third author checked their results (SMP). Each review was assessed by two authors (ACD and LCS). Any disagreements over inclusion, scores, or criteria were settled by consensus amongst the three authors (ACD, LCS and SMP). Each review was given an AMSTAR score which ranged from 1 to 11 (Table 1). The results were then used to determine standardized effectiveness statements (Table 2).

**Table 1:** A measurement tool to assess systematic reviews (AMSTAR) scores.

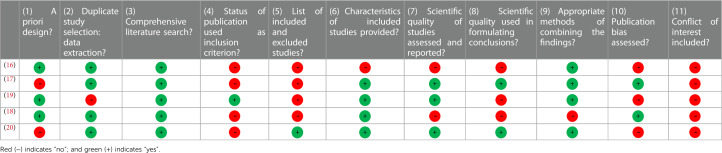

**Table 2:** Summary of reviews investigating the relationship between menstrual cycle phase and acute performance.

Reference	SES	AMSTAR	QoE	Conclusion	Recommendations
Blagrove	Some evidence in favor of no difference	4	3	Findings suggest that strength-related performance is not affected by menstrual cycle phase.	Future research in this field should ensure accurate identification of cycle phases and control for confounding factors that may cause variations in strength performance.
McNulty	Some evidence in favor of no difference	7	3	Results indicate that exercise performance may be trivially reduced during the early follicular phase of the menstrual cycle compared to all other phases.	Due to the trivial effect size, large between-study variability and high quantity of poor-quality studies included in this review, general recommendations could not be made.
Meignie	Some evidence in favor of no difference	6	2	The effect of the menstrual cycle phase on sports performance-related is** **inconclusive.	More high-quality studies that monitor on-field performance parameters are required to enable recommendations for elite women athletes.
Romero-Parra	Sufficient evidence in favor of an effect	5	3	Authors reported that DOMS and strength loss are highest in the EFP and lowest in the MLF, though no significant differences were identified between phases.	Eumenorrheic women should consider lower training loads or longer recovery periods in the EFP and high training loads in the MLP.*
Thompson	Insufficient evidence to determine	7	2	The reviewed articles reported conflicting findings, often limited by small participant numbers and methodological issues, but the authors concluded that women's hormones might affect resistance training responses.	More high-quality experimental studies are needed to understand the effects of the menstrual cycle and oral contraceptives on acute and chronic responses to resistance training.

SES, standardized effectiveness statement; AMSTAR, A Measurement Tool to Assess Systematic Reviews; QoE, quality of evidence.

*We find these recommendations to be unfounded due to high levels of heterogeneity present within the analysis.

The QoE of the included articles ranged from low (QoE = 2) to moderate (QoE = 3), with most reviews being of moderate quality. Three of the five reviews retrieved contained a meta-analysis (16–18).

Blagrove and colleagues analyzed the effect of the menstrual cycle phase on strength-related measures in naturally cycling women (16) (QoE = 3). The meta-analysis included 21 papers with a total of 232 participants. The authors used random-effects meta-analyses to compare strength outcomes between the early-follicular phase (EFP) (defined as days 1–5 of the menstrual cycle; Figure 1) and ovulatory phase (defined as ±2 days post-ovulation); the EFP and mid-luteal phase (MLP) (defined as 7 ± 2 days following ovulation); and the ovulatory phase and MLP. The authors showed that the menstrual cycle phase had a trivial effect on maximal voluntary contraction force, isokinetic peak torque, and explosive strength (Hedges *g* < 0.2). The authors concluded that menstrual cycle phase does not affect strength-related outcomes.

McNulty and colleagues performed a network meta-analysis investigating the effect of menstrual cycle phase on strength and endurance exercise performance (17) (QoE = 3). The review included 73 studies with a total of 954 participants. There were 220 outcome measures included across six phases of the menstrual cycle: the EFP (days 1–5), late follicular phase (LFP) (days 6–12), ovulation (days 13–15), early luteal phase (ELP) (days 16–19), MLP (days 20–23), and late luteal phase (LLP) (days 24–28). All comparisons yielded trivial effect sizes (ES = 0.01–0.14), with the largest difference between the EFP and LFP (ES = 0.14). The authors concluded that exercise performance might be reduced, but to a trivial degree, during the EFP. Due to the almost negligible effect size, the large between-study variability, and the high quantity of poor-quality studies, the authors emphasized that general recommendations could and should not be made.

Meignié et al. performed a systematic review investigating the effect of menstrual cycle phase on exercise performance in elite athletes (19) (QoE = 2). Seven studies with a total of 314 participants were included, six of which reported performance differences between the follicular phase and the luteal phase, but the direction of the differences was inconsistent, and most were not statistically significant. Further, Meignié and colleagues did not define menstrual cycle phase to ensure appropriate comparisons between papers. The authors concluded that, given the inconsistent results, lack of significant findings, and variability in performance outcomes, there is not a clear optimal phase of the menstrual cycle for performance.

Romero-Parra and colleagues reviewed the effect of the menstrual cycle on exercise-induced muscle damage (EIMD) (18) (QoE = 3). Their analysis included 19 studies with a total of 226 participants. Standardized mean differences were used to express differences in muscle soreness and strength from baseline and post-exercise during each of the following phases: EFP (days 2–7), LFP (days 9–13), and MLP (days 18–24). The authors compared the “maximum” mean differences between phases, identifying the largest change in delayed-onset muscle soreness (DOMS) and strength loss during the EFP and the smallest change during the MLF. These authors concluded that DOMS and strength loss are highest in the EFP and lowest in the MLP. Unlike the previous reviews, Romero-Parra and colleagues recommend that “lower training loads or longer recovery periods could be considered in the EFP” and “strength conditioning loads could be enhanced in the MLP”.

Finally, Thompson and colleagues published a systematic review including 17 studies with 418 participants (20) (QoE = 2). Of the 17 studies, only four looked at acute hormonal responses to resistance training in naturally cycling women (51 participants). The remaining articles compared oral contraceptive (OC) users to non-OC users or looked at the effects of phase-based training on chronic adaptations to RET. Despite acknowledging conflicting findings in the reviewed papers, Thompson and colleagues combined the results of several heterogeneous studies (differing study designs, interventions, and subject inclusion criteria) and concluded that women's hormones might affect resistance training responses.

## 3. Discussion

The current umbrella review found scant low-quality and largely inconsistent evidence of marked differences between menstrual cycle phases in strength, exercise performance, and hypertrophy. There may be some evidence suggesting trivial-to-small effects of menstrual cycle phase on indirect markers of DOMS, but the validity of the results is questionable.

### 3.1. Importance of comprehensive menstrual cycle phase detection methods

**Figure 2:**
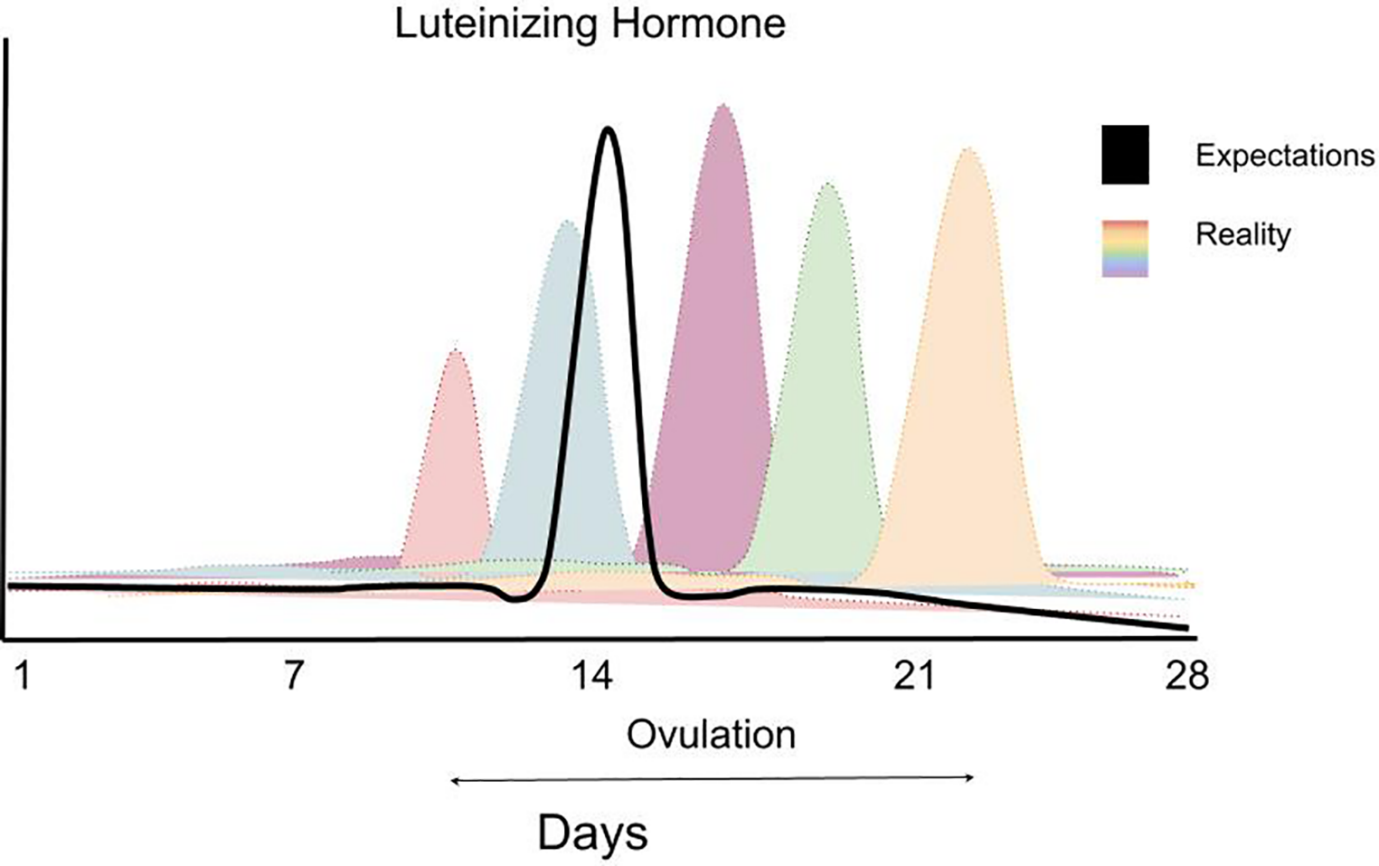
A schematic figure highlighting the variability in LH (luteinizing hormone) surge in women highlighting the impracticality of planning greater training volume in one versus another phase of a woman's menstrual cycle. Data are theoretical but based on data showing the extraordinary variability of the LH surge in women—for example: https://www.ncbi.nlm.nih.gov/pmc/articles/PMC9473716/ (Supplementary Figure S3). Data redrawn with permission.

**Figure 3:**
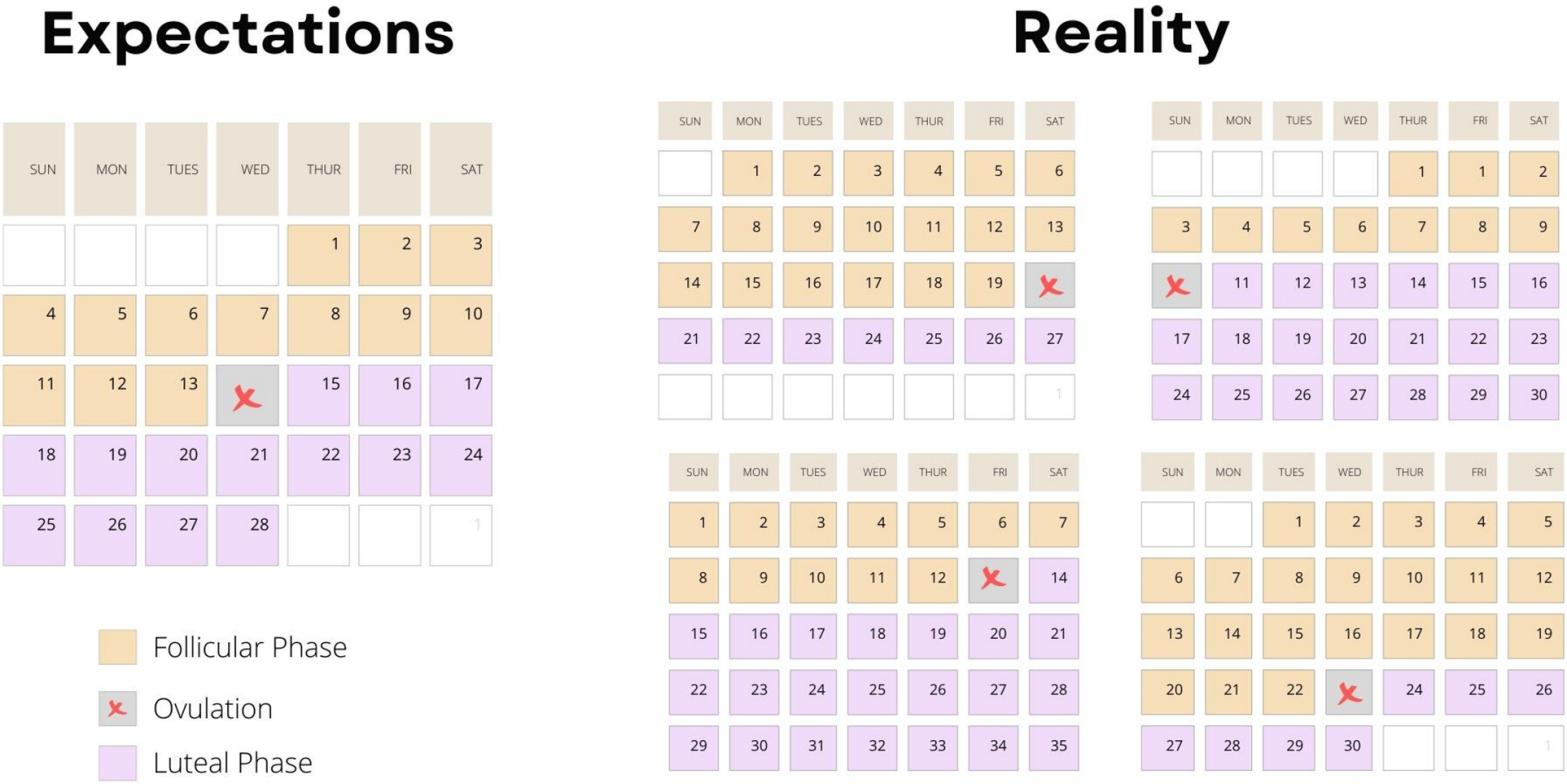
A schematic figure highlighting the impracticality of planning greater training volume in one versus another phase of a woman's menstrual cycle based on data showing the extraordinary variability of the timing of ovulation in women—for example: https://www.ncbi.nlm.nih.gov/pmc/articles/PMC9473716/ (Supplementary Figure S3).

The implementation of menstrual cycle phase-based training is predicated on determining each participant's menstrual cycle phase. Ovulation, characterized by a surge in luteinizing hormone, divides the menstrual cycle into a pre- and post-ovulatory phase, commonly referred to as the follicular and luteal phases, respectively (Figure 1). The follicular phase can range from ∼10 to 22 days, and the luteal phase can range from ∼7 to 17 days (3) (Figure 2). In 1926, leading gynecologist Ludwig Fränkel stated that “ … the only regularity of the menstrual cycle is its irregularity.” (25). Hence, it is unfortunate that, nearly 100 years later, we continue to rely on the assumption that women have repeated 28-day menstrual cycles and ovulate mid-cycle on day 13. This paradigm is a completely inaccurate generalization (Figure 3) that, if assumed, as it was in several key studies in this area (16, 17), results in questionable findings. A resistance exercise prescription based on this assumption is an arbitrary implementation of biweekly undulating periodization, not menstrual cycle “phase-based training”.

Given the variability between individuals and, in some cases, between menstrual cycles (Figures 2, 3) within the same individual, comprehensive menstrual cycle phase detection methods are critical. The most common menstrual cycle phase-detection methods in exercise training research include urinary luteinizing hormone (LH) tests and basal body temperature (BBT) monitoring (25). A luteinizing hormone (LH) surge, which stimulates ovulation, can occur within one day or over 2–6 days (Figure 2), depending on the individual (26). The LH surge concentration in urine ranges from 20 to 100 mIU/ml, and highly sensitive urinary LH kits can detect concentrations as low as 22 mIU/ml (26). This urinary detection method is advantageous as it is non-invasive, inexpensive, and highly accurate.

The thermogenic effect of increased progesterone during the luteal phase can cause a small increase (0.5–1.0°F or 0.3–0.6°C) in basal body temperature (BBT) in some women. It has been assumed that this shift can indicate, retrospectively, that ovulation has occurred. Sung and colleagues utilized this method, stating that “ … the occurrence of ovulation was defined when an increase in basal body temperature of at least 0.3°C was measured.” (21) While this method is non-invasive and convenient, its validity and reliability are questionable; thus, it is not recommended for establishing ovulation (26). Importantly, some women experience ovulation without a clear rise in BBT. For example, Bauman and colleagues evaluated BBT in 98 women and reported that elevated BBT coincided with the LH surge in only 22% of menstrual cycles (27). Additionally, reliable BBT measurements are difficult to obtain, as BBT can be affected by stress, sleep disturbances, illness, alcohol consumption, and environmental factors (28).

Menstrual cycle history and the “calendar counting method” are useful for a general prediction of the onset of menstruation and confirmation of cycle regularity, thus defining women as naturally menstruating rather than eumenorrheic has other associated criteria, such as ovulation and the mid-luteal peak in progesterone. Ideally, research subjects should provide researchers with cycle start dates for at least three, but ideally six, months prior to study enrollment (29). Average cycle lengths are often used to predict the timing of ovulation, but extrapolating any information about phase length incorrectly assumes a correlation between cycle length and the timing of ovulation (30). Moreover, regular menstruation cannot be used to assume that ovulation has occurred (i.e., women can have anovulatory cycles but maintain regular menses) or that luteal phase deficiency is not present (i.e., women can menstruate in the absence of the mid-luteal peak in progesterone). Thus, cycle history should only be used to confirm cycle regularity, and ovulation should be confirmed with a urine or blood test (31).

### 3.2. Does menstrual cycle phase influence acute strength performance? A critical analysis

Three of the five reviews concluded that menstrual cycle phase does not affect strength performance (16, 19, 25), and, in all cases, the authors emphasized the prevalence of low-quality studies, poor methodological practices, and small sample sizes. McNulty and colleagues reported trivial effects of menstrual cycle phase on endurance and strength performance, but only 35 of the 73 studies in the analysis investigated the relationship between menstrual cycle phase and strength outcomes specifically (25). Initially, 22 (63%) of these articles found no significant difference in strength outcomes between menstrual cycle phases; however, some trends were observed when study quality was considered. Most of the papers that found significant differences in strength between phases were ranked low or very low in quality (12/13). When only moderate-to-high-quality studies were included in the analysis, 90% (9/10) of studies found no difference in strength performance between menstrual cycle phases. McNulty's quality assessment was unique because they considered menstrual cycle phase detection methods critical to interpreting study results. Studies that used more robust methods for determining menstrual cycle phases, such as serum hormone levels and urinary ovulation tests, were rated higher in quality than those that did not. Blagrove and colleagues investigated purely strength-related outcome measures and reported a trivial effect of menstrual cycle phase (16). The authors chose to include only studies that used a physiological measure of hormone levels or body temperature to identify or verify menstrual cycle phases, strengthening confidence in their findings. Similarly, Meignié et al. reported that most studies did not yield statistically or practically significant differences in strength performance between menstrual cycle phases in elite athletes (19).

Alternatively, two reviews (18, 20) concluded that menstrual cycle phase might affect resistance training responses. Romero-Parra and colleagues reported that symptoms of DOMS and strength loss were greater in the EFP and lowest in the MLF and recommended considering lower training loads or longer recovery periods in the EFP and high training loads in the MLP based on indirect markers of damage. However, the analysis (18, 20) included very few studies with limited sample sizes (total *n* = 226 and so below the number of subjects considered necessary for a meaningful meta-analysis) and considerable heterogeneity (*I*^2^ > 80%), making it difficult to interpret the results. Until more high-quality studies investigating the effects of menstrual cycle phase on exercise-induced muscle damage, with direct markers of damage, are available, assessments of the literature should be reserved for qualitative analyses. Similarly, Thompson and colleagues reviewed a small number of highly heterogeneous studies; the authors inappropriately combined OC and non-OC users' results to conclude the effect of women's hormones on resistance training adaptations (18, 20). Given that endogenous and exogenous hormones differentially affect women's physiology, it would seem spurious to combine these studies. Thus, the recommendations made by Romero-Parra and Thompson are, in our opinion, premature and potentially misleading.

In sum, the available reviews and meta-analyses suggest that menstrual cycle phase has a limited effect on strength performance and emphasizes the influence of low-quality studies with poor methodological quality on which to base firm conclusions.

### 3.3. Menstrual cycle phase-based training: A critical evaluation

The support for menstrual cycle phase-based resistance training recommendations stems from preliminary evidence from two papers (21, 22). These papers (21, 22) concluded that women could gain muscle strength and size more efficiently by training during the follicular phase, or the first half of the menstrual cycle, compared to luteal phase-based training. Results from one paper even suggested that follicular phase-based training is superior to training regularly throughout the menstrual cycle (22), which is a surprising result given the influence of resistance training volume on hypertrophy. However, several methodological shortcomings of these papers bring into question the validity of their results.

Sung and colleagues concluded that follicular phase-based training was superior to luteal phase-based training for gains in isometric knee extension strength and muscle diameter of the rectus femoris, vastus intermedius, and vastus lateralis (21). The authors reported that their research participants' menstrual cycle length ranged from 25 to 31 days. Subjects trained four times weekly with three supervised sessions of unilateral leg press and one session of unsupervised unilateral bodyweight squats. Follicular phase training started on the first day of the subjects' menstrual cycle, and the transition to luteal phase training was dictated by a basal body temperature increase of at least 0.3°C for 3 days. The training scheme allowed eight training sessions in the follicular phase and only two in the luteal phase, or vice versa. If the subjects completed eight sessions in two weeks, the description implies that every participant ovulated mid-cycle and had symmetrical follicular and luteal phase lengths. Such an occurrence with only 20 subjects seems unlikely given the variability in cycle lengths and use of basal body temperature to detect ovulation. We speculate that using only body temperature as an indirect reflection of ovulatory status is insufficient evidence that these women all trained in the same menstrual cycle phase and that their symmetrical cycles allowed for the same number of sessions in each phase. It is also worth noting that estrogen is cyclical and high in both the follicular and luteal phases, so attributing any effects to this hormone on outcomes in any particular phase would be unclear.

Wikström-Frisén and colleagues also concluded that follicular phase-based training was superior to luteal phase-based training and, surprisingly, was superior to training throughout the full cycle for gains in leg lean mass (22). The training program consisted of leg press and leg curls: 10 sessions in the first two weeks of the cycle and 2 sessions in the last two weeks, or vice versa. All training sessions were unsupervised, allowing for the use of different machines between participants, a lack of standardization in determining load increases, and a lack of consistency in identifying proximity to momentary muscular failure for any particular exercise. Additionally, the training program was designed only for a 28-day cycle and thus assumed that every participant experienced a 14-day follicular phase and a 14-day luteal phase (22). The researchers did not verify actual ovulation timing or menstrual cycle length. Although the sample size of this study was large (*n* = 59), this was a between-group design, which, given the variability in cycle length and phase length between individuals, is problematic. Furthermore, the three groups included naturally cycling participants and participants taking monophasic and triphasic oral contraceptives. Thus, the study cannot be considered a valid comparison of menstrual cycle phase-based training. Finally, the major finding in this paper (22) was that leg lean mass, measured *via* DXA, increased in the follicular phase-based training group but not in the luteal or both phase training groups. The reported change was from 15.1 ± 2.6 kg (mean ± SD) to 15.3 ± 2.7 kg (mean ± SD), a difference that is well within the limit of change in muscle size that DXA can accurately detect in response to exercise (32); however, the authors did not report the test-re-test reliability of their DXA unit so the change cannot be adequately assessed.

A paper by Haines and colleagues (33) is often cited (34) to support the argument that, due to the influence of estradiol and estrogen receptors, post-exercise recovery is faster during the follicular phase of the menstrual cycle. Oddly, the paper does not support this argument. The authors showed significantly greater estrogen receptor alpha (ER-α) mRNA and protein expression in the follicular phase compared to the luteal phase. However, while ER-DNA binding (indicative of ER-mediated gene activation) and Myo-D mRNA expression increased with eccentric exercise, they were not different between menstrual cycle phases. Cyclin D1 mRNA expression was significantly greater during the follicular phase. A cyclin D1-induced ER activation mechanism *can* result in subsequent increases in Myo-D mRNA expression, but the authors did not observe a difference in Myo-D mRNA expression between cycle phases. Haines concluded that “…estradiol seems to play (a role) in the myogenic activation of satellite cells as they assist with muscle repair and regeneration during recovery”. However, there is no evidence to suggest a phase-based difference in this regard, and the conclusion is speculation that is not supported by data.

## 4. Directions for future research

Menstrual cycle length, phase length, and LH surge timing vary greatly between individuals. Given this variability, within-subject designs are more robust for investigating phase-based differences than between-subjects study designs. Cycle length, phase length, and LH surge timing also vary *within* individuals. An analysis of menstrual cycles in 130 women demonstrated variability between cycle length within a person of >7 days (35). Additionally, ovulation and the mid-luteal peak in progesterone does not occur in every cycle for every woman (Figures 2, 3). Thus, phase-specific data should be collected from each participant during each study cycle. These caveats emphasize the importance of using a robust methodological approach to determine menstrual cycle phase. If a protocol did not utilize an accurate and reliable menstrual cycle phase detection method, how can one confidently attribute the study findings to the hormonal profile of the menstrual cycle phase?

A high-quality study of long-term adaptations to menstrual cycle phase-based resistance training should be conducted in eumenorrheic women with a history of minimal menstrual cycle variability (<3 days) between cycle lengths, none of whom are using hormonal contraceptives. Currently, no such study exists. Eumenorrheic status should be determined with urinary ovulation tests and serum estradiol and progesterone tests in both phases. The phase-based training should be tailored to each participant's individual cycle, with RET volume-load (load·repetitions/set·sets/exercise·exercises/session·sessions/week) equated between cycles.

## 5. Conclusions

Premenopausal women are frequently excluded from exercise physiology research to avoid the potential influence of varying ovarian hormones across the menstrual cycle. This assumption perpetuates a widespread sex-based bias in the exercise physiology literature (4, 36), despite profound interindividual variability in the magnitude of hypertrophic response to RET in both women and men (37, 38). Further, we note that detailed comparisons of men and women in their propensity for RET-induced hypertrophy indicate equivalent relative gains in muscle size and, for the most part, strength (39). In the absence of high-quality evidence to indicate that cyclical hormonal fluctuations substantially influence acute strength performance or RET-induced muscular adaptations, it is, in our view, premature to assume that it is essential to control for the menstrual cycle phase in which women are tested.

## 6. Practical recommendations

In the absence of high-quality evidence to support designing resistance training programs based on menstrual cycle phase, coaches and athletes should tailor an exercise plan to the individual. The influence of the menstrual cycle could be a factor to consider in program design, along with a host of other factors: nutrition, fatigue, sleep quality, stress, injury, motivation, and program enjoyment. We acknowledge that menstrual symptoms can influence exercise performance in some women (40), and thus it would be helpful to document this for reviewing long-term progress and adjusting a program. When reviewing the evidence as a whole—and the methodological shortcomings therein—we propose it is highly premature to conclude that short-term fluctuations in ovarian hormones appreciably influence acute exercise performance or longer-term adaptations to resistance training. Thus, the development of RET prescriptions based on cyclical hormonal changes is not an evidence-based approach.

## Data Availability

The original contributions presented in the study are included in the article/Supplementary Material, further inquiries can be directed to the corresponding author.

## References

[1] Lim C Nunes EA Currier BS McLeod JC Thomas ACQ Phillips SM. An evidence-based narrative review of mechanisms of resistance exercise-induced human skeletal muscle hypertrophy. Med Sci Sports Exerc. (2022) 54(9):1546–59. 10.1249/MSS.0000000000002929 35389932 PMC9390238

[2] Smith GI Mittendorfer B. Sexual dimorphism in skeletal muscle protein turnover. J Appl Physiol. (2016) 120(6):674–82. 10.1152/japplphysiol.00625.2015 26702024 PMC4796180

[3] Shea A Vitzthum V. The extent and causes of natural variation in menstrual cycles: integrating empirically-based models of ovarian cycling into research on women’s Health. Drug Discov Today Dis Models. (2020) 32:41–9. 10.1016/j.ddmod.2020.11.002

[4] Smith ES McKay AKA Kuikman M Ackerman KE Harris R Elliott-Sale KJ Auditing the representation of female versus male athletes in sports science and sports medicine research: evidence-based performance supplements. Nutrients. (2022) 14(5):953. 10.3390/nu14050953 PMC8912470 35267928

[5] Areta JL Elliott-Sale KJ. Nutrition for female athletes: what we know, what we don't know, and why. Eur J Sport Sci. (2022) 22(5):669–71. 10.1080/17461391.2022.2046176 35195492

[6] Costello JT Bieuzen F Bleakley CM. Where are all the female participants in sports and exercise medicine research? Eur J Sport Sci. (2014) 14(8):847–51. 10.1080/17461391.2014.911354 24766579

[7] Morton RW Oikawa SY Wavell CG Mazara N McGlory C Quadrilatero J Neither load nor systemic hormones determine resistance training-mediated hypertrophy or strength gains in resistance-trained young men. J Appl Physiol. (2016) 121(1):129–38. 10.1152/japplphysiol.00154.2016 27174923 PMC4967245

[8] Enns D Tiidus P. The influence of estrogen on skeletal muscle. Sports Med. (2010) 40(1):41–58. 10.2165/11319760-000000000-00000 20020786

[9] Ikeda K Horie-Inoue K Inoue S. Functions of estrogen and estrogen receptor signaling on skeletal muscle. J Steroid Biochem Mol Biol. (2019) 191:105375. 10.1016/j.jsbmb.2019.105375 31067490

[10] Chidi-Ogbolu N BK. Effect of estrogen on musculoskeletal performance and injury risk. Front Physiol. (2019) 9:1834. 10.3389/fphys.2018.01834 30697162 PMC6341375

[11] Collins BC Arpke RW Larson AA Baumann CW Xie N Cabelka CA Estrogen regulates the satellite cell compartment in females. Cell Rep. (2019) 28:368–81. 10.1016/j.celrep.2019.06.025 31291574 PMC6655560

[12] Lamont LS LP Bruot BC. Menstrual cycle and exercise effects on protein catabolism. Med Sci Sports Exerc. (1987) 19(2):106–10. 3574042

[13] Heikkinen J KE Kurttila-Matero E Wilén-Rosenqvist G Lankinen KS Rita H Väänänen HK. HRT And exercise: effects on bone density, muscle strength and lipid metabolism. A placebo controlled 2-year prospective trial on two estrogen-progestin regimens in healthy postmenopausal women. Maturitas. (1997) 26(2):139–49. 10.1016/S0378-5122(96)01098-5 9089564

[14] Collins BC LE Lowe DA. Aging of the musculoskeletal system: how the loss of estrogen impacts muscle strength. Bone. (2019) 123:137–44. 10.1016/j.bone.2019.03.033 30930293 PMC6491229

[15] Smith GI Reeds DN Hall AM Chambers KT Finck BN Mittendorfer B. Sexually dimorphic effect of aging on skeletal muscle protein synthesis. Biol Sex Differ. (2012) 3:11. 10.1186/2042-6410-3-11 22620287 PMC3467175

[16] Blagrove RC Bruinvels G Pedlar CR. Variations in strength-related measures during the menstrual cycle in eumenorrheic women: a systematic review and meta-analysis. J Sci Med Sport. (2020) 23(12):1220–7. 10.1016/j.jsams.2020.04.022 32456980

[17] McNulty KL Elliott-Sale KJ Dolan E Swinton PA Ansdell P Goodall S The effects of menstrual cycle phase on exercise performance in eumenorrheic women: a systematic review and meta-analysis. Sports Med. (2020) 50(10):1813–27. 10.1007/s40279-020-01319-3 32661839 PMC7497427

[18] Romero-Parra N Cupeiro R Alfaro-Magallanes VM Rael B Rubio-Arias JÁ Peinado AB Exercise-Induced muscle damage during the menstrual cycle: a systematic review and meta-analysis. J Strength Cond Res. (2021) 35(2):549–61. 10.1519/JSC.0000000000003878 33201156

[19] Meignié A Duclos M Carling C Orhant E Provost P Toussaint JF The effects of menstrual cycle phase on elite athlete performance: a critical and systematic review. Front Physiol. (2021) 12:654–585. 10.3389/fphys.2021.654585 PMC8170151 34093223

[20] Thompson B Almarjawi A Sculley D Janse de Jonge X. The effect of the menstrual cycle and oral contraceptives on acute responses and chronic adaptations to resistance training: a systematic review of the literature. Sports Med. (2020) 50(1):171–85. 10.1007/s40279-019-01219-1 31677121

[21] Sung E Han A Hinrichs T Vorgerd M Manchado C Platen P. Effects of follicular versus luteal phase-based strength training in young women. SpringerPlus. (2014) 3:668. 10.1186/2193-1801-3-668 25485203 PMC4236309

[22] Wikström-Frisén L Boraxbekk CJ Henriksson-Larsén K. Effects on power, strength and lean body mass of menstrual/oral contraceptive cycle-based resistance training. J Sports Med Phys Fitness. (2017) 57:43–52. 10.23736/S0022-4707.16.05848-5 26558833

[23] Shea BJ Reeves BC Wells G Thuku M Hamel C Moran J AMSTAR 2: a critical appraisal tool for systematic reviews that include randomised or non-randomised studies of healthcare interventions, or both. Br Med J. (2017) 358:j4008. 10.1136/bmj.j4008 PMC5833365 28935701

[24] Mustafa RA Santesso N Brozek J Akl EA Walter SD Norman G Kulasegaram M The GRADE approach is reproducible in assessing the quality of evidence of quantitative evidence syntheses. J Clin Epidemiol. (2013) 66(7):736–42. 10.1016/j.jclinepi.2013.02.004 23623694

[25] Vitzthum V. Field methods and strategies for assessing female reproductive functioning. Am J Hum Biol. (2021) 33:e23513. 10.1002/ajhb.23513 33022128

[26] Su HW Yi YC Wei TY Chang TC Cheng CM. Detection of ovulation, a review of currently available methods. Bioeng Transl Med. (2017) 2(3):238–46. 10.1002/btm2.10058 29313033 PMC5689497

[27] B JE. Basal body temperature: unreliable method of ovulation detection. Fertil Steril. (1981) 36(6):729–33. 10.1016/S0015-0282(16)45916-9 7308516

[28] Barron ML Fehring RJ. Basal body temperature assessment: is it useful to couples seeking pregnancy? MCN Am J Matern Child Nurs. (2005) 30(5):290–6. 10.1097/00005721-200509000-00004 16132004

[29] Elliott-Sale KJ Minahan CL de Jonge XAKJ Ackerman KE Sipilä S Constantini NW Methodological considerations for studies in sport and exercise science with women as participants: a working guide for standards of practice for research on women. Sports Med. (2021) 51(5):843–61. 10.1007/s40279-021-01435-8 33725341 PMC8053180

[30] Johnson S Marriott L Zinaman M. Can apps and calendar methods predict ovulation with accuracy? Curr Med Res Opin. (2018) 34(9):1587–94. 10.1080/03007995.2018.1475348 29749274

[31] Janse DJX Thompson B Han A. Methodological recommendations for menstrual cycle research in sports and exercise. Med Sci Sports Exerc. (2019) 51(12):2610–7. 10.1249/MSS.0000000000002073 31246715

[32] Tavoian D Ampomah K Amano S Law TD Clark BC. Changes in DXA-derived lean mass and MRI-derived cross-sectional area of the thigh are modestly associated. Sci Rep. (2019) 9(1):10028. 10.1038/s41598-019-46428-w 31296891 PMC6624257

[33] Haines M McKinley-Barnard SK Andre TL Gann JJ Hwang PS Willoughby DS. Skeletal muscle estrogen receptor activation in response to eccentric exercise up-regulates myogenic-related gene expression independent of differing serum estradiol levels occurring during the human menstrual cycle. J Sports Sci Med. (2018) 17:31–9. 29535576 PMC5844207

[34] Kissow J Jacobsen KJ Gunnarsson TP Jessen S Hostrup M. Effects of follicular and luteal phase-based menstrual cycle resistance training on muscle strength and mass. Sports Med. (2022) 52(12):2813–19. 10.1007/s40279-022-01679-y 35471634

[35] Creinin M Keverline S Meyn L. How regular is regular? An analysis of menstrual cycle regularity. Contraception. (2004) 70(4):289–92. 10.1016/j.contraception.2004.04.012 15451332

[36] Lew LA Williams JS Stone JC Au AKW Pyke KE MacDonald MJ. Examination of sex-specific participant inclusion in exercise physiology endothelial function research: a systematic review. Front Sports Act Living. (2022) 4:860356. 10.3389/fspor.2022.860356 35399599 PMC8990239

[37] Bamman MM Petrella JK Kim JS Mayhew DL Cross JM. Cluster analysis tests the importance of myogenic gene expression during myofibre hypertrophy in humans. J Appl Physiol. (2007) 102:2232–9. 10.1152/japplphysiol.00024.2007 17395765

[38] Roberts MD Haun CT Mobley CB Mumford PW Romero MA Roberson PA Physiological differences between low versus high skeletal muscle hypertrophic responders to resistance exercise training: current perspectives and future research directions. Front. Physiol. (2018) 9:834. 10.3389/fphys.2018.00834 30022953 PMC6039846

[39] Roberts BM Nuckols G Krieger JW. Sex differences in resistance training: a systematic review and meta-analysis. J Strength Cond Res. (2020) 34(5):1448–60. 10.1519/JSC.0000000000003521 32218059

[40] Ekenros L von Rosen P Solli GS Sandbakk Ø Holmberg HC Hirschberg AL Perceived impact of the menstrual cycle and hormonal contraceptives on physical exercise and performance in 1,086 athletes from 57 sports. Front Physiol. (2022) 13:954760. 10.3389/fphys.2022.954760 36111164 PMC9468598

